# The Association between Polypharmacy and Dementia: A Nested Case-Control Study Based on a 12-Year Longitudinal Cohort Database in South Korea

**DOI:** 10.1371/journal.pone.0169463

**Published:** 2017-01-05

**Authors:** Hae-Young Park, Ji-Won Park, Hong Ji Song, Hyun Soon Sohn, Jin-Won Kwon

**Affiliations:** 1 College of Pharmacy and Research Institute of Pharmaceutical Sciences, Kyungpook National University, Daegu, Korea; 2 College of Natural Science, Kyungpook National University, Daegu, Korea; 3 Department of Family Medicine, Hallym University Sacred Heart Hospital, Gyeonggi-do, Korea; 4 Graduate School of Clinical Pharmacy, CHA University, Gyeonggi-do, Korea; Universidade Federal do Rio de Janeiro, BRAZIL

## Abstract

Dementia is a major concern among growing chronic diseases in the aging society and its association with polypharmacy has not been adequately assessed. The objective of this study was to determine the association between polypharmacy and dementia through multiple statistical approaches. We conducted a nested case-control study for newly diagnosed dementia cases using the South Korean National Health Insurance Service sample cohort database (2002–2013, n = 1,025,340). Interactions between polypharmacy (an average use of ≥5 prescription drugs daily) and comorbidities or potentially inappropriate medications (PIMs) were tested. The odds ratios (ORs) for dementia were analyzed according to the presence of comorbidities, PIM uses, the average number of prescribed daily drugs, and significant interactions with polypharmacy using univariate and multiple logistic regression analyses. A higher prevalence of comorbidities, history of PIM use, higher PIM exposure, and higher proportion of polypharmacy were noted among cases than in controls. In the univariate analysis, the OR for dementia increased significantly with the increase in the number of prescribed drugs [1–<5 drugs: 1.72, 95% confidence interval (CI): 1.56–1.88; 5–<10 drugs: 2.64, 95% CI: 2.32–3.05; ≥10 drugs: 3.35, 95% CI: 2.38–4.71; <1 drug used as reference]. Polypharmacy was correlated with comorbidities and PIM use, and significant interactions were observed between polypharmacy and anticholinergics; H2-receptor antagonists; and comorbidities such as hypertension, peripheral or cerebrovascular disease, congestive heart failure, hemiplegia, diabetes, depression, all other mental disorders, chronic obstructive pulmonary disease, peptic ulcer disease, and chronic liver disease (p<0.001). In the multiple regression analysis, most cases exhibited increasing ORs for dementia with increasing polypharmacy levels. Moreover, the increase in OR was more evident in the absence of drugs or comorbidities that showed significant interactions with polypharmacy than in their presence. Polypharmacy increases the risk of PIM administration, and as some PIMs may have cognition-impairing effects, prolonged polypharmacy may result in dementia. Therefore, efforts are needed to limit or decrease the prescription of medications that have been associated with risk of dementia in the elderly.

## Introduction

By the year 2050, approximately 1.5 billion people worldwide would be aged ≥65 years; nearly triple the number reported in 2010. This increase in the elderly population, accompanied by an increase in chronic diseases and polypharmacy, is a major global public health concern facing our generation [1]. In the Republic of Korea, the elderly (aged ≥65 years) will comprise more than one-third of the total population by 2050. Further, the proportion of patients with chronic diseases amongst the elderly population has increased from 85.5% in 2008 to 93.9% in 2011, and the average number of comorbidities per patient has increased from 2.7 to 4.2 [2]. These trends inevitably lead to both the overall rise in health care costs and declines in the health-related quality of life [3]. Dementia, due to its rapidly increasing incidence and a high economic burden as a result of the growing social care and direct treatment-related costs to the patients, is one of the chronic diseases whose management has received very high priority. By 2050, the number of people with dementia is expected to be more than triple of what it is today [4], and as of 2010, the worldwide dementia management cost is estimated at USD 604 billion, an amount likely to increase further in the future [5].

Increase in the number of elderly people, and in the incidence of chronic diseases are both linked to polypharmacy, which is defined by the world health organization as the administration of multiple drugs concurrently or an excessive number of drugs [6]. However, as the definition of polypharmacy does not specify the duration of administration and the number of drugs, most commonly used criterion is the administration of five or more drugs per day for a certain period of time [7–12]. Polypharmacy and incidence of chronic diseases have increased concomitantly. In the elderly, prolonged exposure to polypharmacy results in a vicious cycle wherein the treatment of chronic diseases results in polypharmacy, which in turn gives rise to new comorbidities requiring further medication [13, 14]. Furthermore, taking a number of drugs simultaneously may increase the risk of transient adverse drug reactions, and the patients’ health outcomes may deteriorate irreversibly with extended periods of polypharmacy [15–18].

Previous studies have demonstrated that polypharmacy increases the risk of unfavorable health outcomes such as adverse drug reactions, falls, fall-related outcomes, increased frequency of hospitalization, and mortality [9, 11]. However, only a few studies have assessed the association between polypharmacy and dementia. Lau *et al*. studied the association between polypharmacy and potentially inappropriate medication (PIM) use in elderly people (≥65 years) with dementia using a 4-year observational dataset from the National Alzheimer's Coordinating Center in the United States. Their study noted that elderly people receiving five or more medications exhibited a higher tendency for PIM administration than those receiving less than five drugs [19]. In the study by Lai *et al*., polypharmacy, which they defined as the administration of five or more drugs, was observed at significantly higher frequency in the dementia-afflicted cases than in the controls (P<0.00001). Moreover, they noted that the increasing number of drugs and age were both associated with the incidence of dementia [20]. However, the study by Lau *et al*. could not evaluate the causal relationship between dementia and polypharmacy due to the cross-sectional study design, and the study by Lai *et al*. did not consider the complex interactions between PIMs and/or comorbidities and polypharmacy. Therefore, this study aimed to analyze the association between polypharmacy and dementia in a large patient cohort database. We utilized a 12-year longitudinal cohort (2002–2013), representing the entire population of South Korea, to evaluate the association between polypharmacy and dementia, taking into account the interaction among PIMs and comorbidities. Our study results emphasize the need for effective management of polypharmacy and drug administration to reduce the risk of dementia in the elderly.

## Methods

### Study database

The National Health Insurance Service-National Sample Cohort (NHIS-NSC) database (n = 1,025,340; 2002–2013) was used for this study. The South Korean NHIS system includes the entire national population (~50 million people), and the database was established for claim reimbursements. The NHIS-NSC database was constructed by the NHIS for health-related research, and the data is openly accessible to researchers. For sampling, 756 strata based on some variables such as 18 groups for age, 21 groups for income level by insurance type (10 groups for NHIS district subscriber and 10 groups for NHIS employee subscriber, one group for medical aid), and sex was set up by using 2002 data. The sampled individuals were followed until 2013. As a semi-dynamic cohort, information on newborns was added to the NHIS-NSC database to supplement data points lost due to deaths [21]. Data on subject characteristics, clinical information, beneficiary’s socioeconomic level, and death records were included in the database. Clinical information including disease diagnosis codes based on the International Codes of Disease 10^th^ Edition (ICD-10) Clinical Modification, treatments based on drug prescriptions, and health care costs were recorded.

### Patient involvement

Patients were not directly involved in the research, and only the secondary electronic database was used for the analysis. Informed consent was not required as the database maintained de-identification and anonymity of sampled individuals. This study was approved by the Kyungpook National University Institutional Review Board (KNU 2014–85–0).

### Study design and selection of cases and controls

A nested case-control design was applied to this study. Community-dwelling elderly patients aged ≥65 years with no prior hospitalization history were initially selected from the NHIS-NSC database. Cases were defined as community-dwelling elderly patients with dementia who utilized outpatient service more than once, who had no prior hospitalization history, and who were newly diagnosed with dementia from 2005 to 2013. Dementia was defined as the presence of more than one of the following ICD-10 codes: F00 (dementia in Alzheimer’s disease), F01 (vascular dementia), F02 (dementia in other diseases classified elsewhere), F03 (unspecified dementia), F051 (delirium superimposed on dementia), G30 (Alzheimer’s disease), and G311 (senile degeneration of the brain, not elsewhere classified). Controls were selected through a 1:1 matching to the cases based on age, sex, index of income level, and year of dementia diagnosis.

### Polypharmacy prescription

The index date was set as the date of dementia diagnosis in a case, and the matched control had the same index date as the case. Drug prescription history was investigated for the look-back period, defined as the last 2 years from the index date. The number of average prescribed daily drugs were estimated by adding all drugs prescription days in the 2-year period and dividing the sum by 730, a formula based on a previous study by Lai *et al*. (average number of prescribed daily drugs = {[(1 type of drug) × (days used)] + [(2 types of drug) × (days used)] + [(3 types of drug) × (days used)] + [(4 types of drug) × (days used)] + [(5 types of drug) × (days used) +… + [(10 or more types of drug) × (days used)]/730 days). In the case of a combination of drugs for hypertension or diabetes, the number of active ingredients were used for calculations instead of the number of drugs, and polypharmacy was defined as an average prescription of five or more drugs per day [20].

### Comorbidities

The Charlson comorbidity index (CCI) diseases, and a range of comorbidities related to dementia, such as hypertension, depression, and psychiatric diseases, were compared between the cases and controls during the look-back period. The CCI scores were used as health status indices and were calculated according to the prevalence of CCI diseases [22]. All comorbidities were defined by the ICD-10 codes, and the list of diseases and the ICD-10 codes used for the analysis are summarized in S1 Table.

### Potentially inappropriate medication (PIM)

Based on the 2015 American Geriatrics Society Beers Criteria, PIM for dementia includes benzodiazepines, anticholinergic drugs, and H2-receptor antagonists [23]. PIM use was thus defined as the prescription for benzodiazepines, anticholinergic drugs, and H2-receptor antagonists during the look-back period. PIM use was measured according to the medication procession ratio (MPR), which is defined for each specific PIM as the percentage of prescription days for the total observation period (i.e., 2 years for this study).

### Statistical analysis

The demographic and clinical characteristics of the study population were analyzed and described by average and standard deviation (SD) for numerical variables, and frequency and percentage were used for categorical variables. The association between the use of PIMs and the diagnosis of dementia was analyzed using a univariate logistic regression analysis. To estimate the association between polypharmacy and dementia, crude odds ratios (ORs) were calculated using univariate logistic regression analyses for total subjects and various subgroups. Subject subgroups were defined based on the type of dementia, or the presence/absence of comorbidities. Dementia patients were classified into three categories: patients with Alzheimer’s disease with ICD-10 codes of F00 and G30, without F01, F02, F03, F051, G311; patients with other causes of dementia with ICD-10 codes of F01, F02, F03, F051, and G311, without ICD-10 codes of F00 or G30; and mixed dementia patients with Alzheimer’s disease codes (F00 or G30) and any other causes of dementia codes (F01, F02, F03, F051, G311) simultaneously. The univariate logistics regression analyses for comorbidity-based patient subgroups were integrated by inverse variance and are presented as integrated ORs. Interactions between polypharmacy and comorbidities, or PIM, were tested, and multiple logistic regression analyses were performed to adjust for comorbidity status and PIM use with or without the inclusion of interactions between polypharmacy and comorbidities or PIMs.

## Results

**Fig 1:**
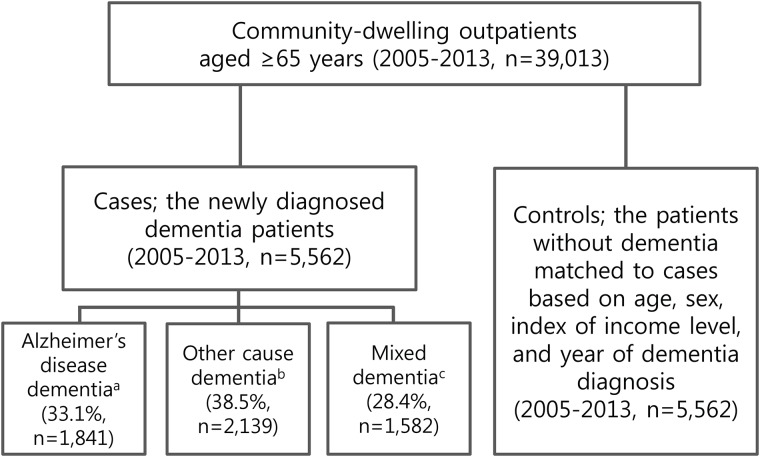
Selection of cases and controls. ^a^ Alzheimer’s disease dementia: patients with ICD-10 codes of F00 and G30, without F01, F02, F03, F051, and G311. ^b^ Other cause dementia: patients with ICD-10 codes of F01, F02, F03, F051, and G311, without ICD-10 codes of F00 or G30 ^c^ Mixed dementia: patients with Alzheimer’s disease codes (F00 or G30) and any other cause dementia codes (F01, F02,F03, F051, G311) simultaneously.

The total number of community-dwelling outpatients aged ≥65 years, without any hospitalization history, was 39,013. Of them, a total of 5,562 individuals fulfilled the inclusion criteria for the cases and controls. The proportions of dementia subtypes with Alzheimer’s disease, other causes, and mixed type of both Alzheimer’s disease and other causes were 33.1% (n = 1,841), 38.5% (n = 2,139), and 28.4% (n = 1,582), respectively (Fig 1).

The study population comprised 71.8% women, and the average age of the population was 73.3 (SD: 6.9) years. Cases had 2.14-point higher CCI score compared to controls (3.65 versus 1.51). Comorbidities were more prevalent in the cases. The prevalence of cerebrovascular diseases, hemiplegia, depression, delirium, behavioral disorders due to alcohol, schizophrenia, and psychotic disorders were >3-times higher in the cases than in controls. Hypertension was the most common comorbidity noted in both groups, and its prevalence was higher in the cases than in the controls (66.6% versus 48.6%). In general, the other cause dementia cases exhibited a higher prevalence of comorbidities than the Alzheimer’s disease and mixed dementia cases (S2 Table). The average number of prescribed daily drugs during the look-back period was 2.54 (SD: 2.87) for the cases and 1.75 (SD: 2.39) for the controls. Thus, the subjects in the case group were administered 0.79 drugs more each day during the 2 years prior to dementia diagnosis, compared to the subjects in the control group (Table 1).

**Table 1:** Demographic and clinical information for cases and controls.

Number of subjects		Cases (n = 5,562)		Controls (n = 5,562)	
		n	(%)	n	(%)
**Sex**^a^	Male	1,566	(28.2)	1,566	(28.2)
	Female	3,996	(71.8)	3,996	(71.8)
**Age**^a^	Mean [SD]	73.3	[6.9]	73.3	[6.9]
	65–<75 years	2,812	(50.6)	2,812	(50.6)
	≥75 years	2,750	(49.4)	2,750	(49.4)
**CCI score**^b^	Mean [SD]	3.65	[2.24]	1.51	[1.86]
**CCI diseases**^b^	Myocardial infarction	107	(1.9)	58	(1.0)
	Congestive heart failure	572	(10.3)	322	(5.8)
	Peripheral vascular disease	1,492	(26.8)	831	(14.9)
	Cerebrovascular disease	2,110	(37.9)	591	(10.6)
	Dementia	5,562	(100.0)	0	(0.0)
	Chronic obstructive pulmonary disease	2,025	(36.4)	1,500	(27.0)
	Connective tissue disease	351	(6.3)	184	(3.3)
	Peptic ulcer disease	1,863	(33.5)	1,272	(22.9)
	Chronic liver disease	1,274	(22.9)	792	(14.2)
	Diabetes mellitus (uncomplicated)	1,559	(28.0)	978	(17.6)
	Diabetes mellitus (complicated)	664	(11.9)	394	(7.1)
	Hemiplegia	234	(4.2)	43	(0.8)
	Moderate/severe kidney disease	94	(1.7)	58	(1.0)
	Tumor, leukemia, lymphoma	522	(9.4)	319	(5.7)
	Moderate/severe liver disease	27	(0.5)	12	(0.2)
	Metastatic solid tumor	46	(0.8)	32	(0.6)
	Acquired immune deficiency syndrome	1	(0.0)	0	(0.0)
**Other comorbidities related with dementia**^b^	Hypertension	3,702	(66.6)	2,702	(48.6)
	Depression	959	(17.2)	326	(5.9)
	Delirium	67	(1.2)	4	(0.1)
	Behavior disorders due to alcohol	43	(0.8)	11	(0.2)
	Schizophrenia/psychotic disorders	108	(1.9)	8	(0.1)
	All other mental disorders	2,500	(44.9)	1,309	(23.5)
**Average number of drugs**^c^	Mean [SD]	2.54	[2.87]	1.75	[2.39]

CCI: Charlson comorbidity index, DM: diabetes mellitus, SD: standard deviation.

^a^ The case and control groups had same distribution for age and sex due to matching.

^b^ CCI score, CCI disease, and other comorbidities were analyzed for the look-back period (0–2 years prior to the index date).

^c^ The number of average prescribed daily drugs were estimated by adding all drugs prescription days in the look-back period and dividing the sum by 730.

**Fig 2:**
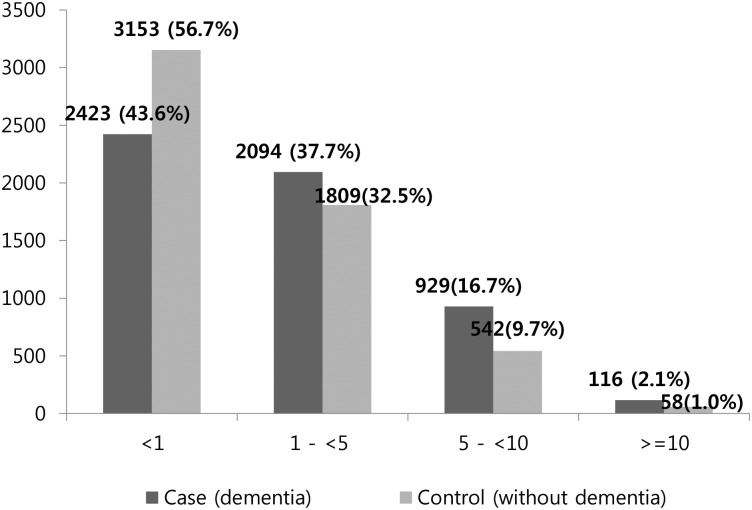
Prevalence of polypharmacy in cases and controls.

The prevalence of polypharmacy was 1.7-times more in cases; polypharmacy was observed in 18.8% of the cases and in 10.8% of the controls (Fig 2).

The OR of a PIM prescription was 1.5–2.0 in the cases, indicating that the cases had a significantly higher exposure to PIMs than controls. The univariate OR for dementia increased with longer duration of PIM use (number of PIM prescription days) (Table 2).

**Table 2:** Exposure to dementia and cognitive impairment-related potentially inappropriate medications in cases and controls.

PIM	MPR^a^	Cases (n = 5,562)	Controls (n = 5,562)	OR (95% CI)	MPR^a^	Cases (n = 5,562)	Controls (n = 5,562)	OR (95% CI)
		n (%)	n (%)			n (%)	n (%)	
**Benzodiazepine (all)**	0	2,880 (52)	3,562 (64)	1	0	2,880 (52)	3,562 (64)	1
	>0–100	2,682 (48)	2,000 (36)	1.73 (1.60–1.88)	>0–<50	2,325 (42)	1,854 (33)	1.63 (1.50–177)
					50–100	357 (6)	146 (3)	3.16 (2.58–3.86)
**Benzodiazepine (short or intermediate acting)**	0	3,842 (69)	4,402 (79)	1	0	3,842 (69)	4,402 (79)	1
	>0–100	1,720 (31)	1,160 (21)	1.77 (1.62–1.94)	>0–<50	1,524 (27)	1,066 (19)	1.71 (1.55–1.88)
					50–100	196 (4)	94 (2)	2.46 (1.91–3.16)
**Benzodiazepine (long acting)**	0	3,849 (69)	4,284 (77)	1	0	3,849 (69)	4,284 (77)	1
	>0–100	1,713 (31)	1,278 (23)	1.51 (1.39–1.65)	>0–<50	1,604 (29)	1,233 (22)	1.47 (1.35–1.60)
					50–100	109 (2)	45 (1)	2.75 (1.94–3.90)
**Benzodiazepine (receptor agonist)**	0	5,023 (90)	5,277 (95)	1	0	5,023 (90)	5,277 (95)	1
	>0–100	539 (10)	285 (5)	2.00 (1.72–2.33)	>0–<50	504 (9)	271 (5)	1.97 (1.69–2.30)
					50–100	35 (1)	14 (0)	2.66 (1.43–5.00)
**Anticholinergic drugs**	0	2,445 (44)	3,140 (57)	1	0	2,445 (44)	3,140 (57)	1
	>0–100	3,117 (56)	2,422 (44)	1.77 (1.63–1.92)	>0–<50	2,903 (52)	2,309 (42)	1.73 (1.59–1.87)
					50–100	214 (4)	113 (2)	2.63 (2.07–3.35)
**H2-receptor antagonist**	0	2,381 (43)	2,964 (53)	1	0	2,381 (43)	2,964 (53)	1
	>0–100	3,181 (57)	2,598 (47)	1.66 (1.52–1.80)	>0–<50	2,959 (53)	2,472 (44)	1.62 (1.49–1.76)
					50–100	222 (4)	126 (2)	2.47 (1.96–1.76)

PIM: potentially inappropriate medication, OR: odds ratio, CI: confidence interval.

^a^ MPR: medication procession ratio, MPR was defined as the percentage of prescription days for the 2-year look-back period.

Univariate logistic regression analysis revealed that polypharmacy was associated with higher OR for dementia. The ORs for dementia increased significantly with the increasing number of prescribed drugs (1–<5 drugs: 1.72, 95% CI: 1.56–1.88; 5–<10 drugs: 2.64, 95% CI: 2.32–3.05; and ≥10 drugs: 3.35, 95% CI 2.38–4.71; <1 drug used as reference). This trend for an increase in dementia OR with an increase in the number of prescribed drugs was stronger for other cause dementia than for Alzheimer’s disease dementia. The integrated OR for the subgroup of patients with no specific comorbidities was 2.61 (95% CI: 2.51–2.72) for 5–<10 used drugs, with the <1 drug group used as a reference. However, the integrated OR was lower for the subgroup of patients with a specific comorbidity: 1.21 (1.07–1.38) for 5–<10 used drugs (Table 3). The individual ORs for comorbidity-based patient subgroups were higher in the subgroup with no specific comorbidity than in the subgroup where comorbidities were present (S3 Table).

**Table 3:** Univariate logistic regression analysis for polypharmacy and dementia segregated by patient subgroups.

Average number of prescribed daily drugs	Crude OR (95% CI)				Integrated ORs^d^ for subgroups with no specific comorbidities	Integrated ORs^d^ for subgroups with specific comorbidities
	Total population	Alzheimer’s disease dementia^a^	Other cause dementia^b^	Mixed dementia^c^		
	(n = 5,562)	(n = 1,841)	(n = 2,139)	(n = 1,582)		
**<1**	1	1	1	1	1	1
**1–<5**	1.72	1.44	2.31	1.49	1.73	0.91
	(1.56–1.88)	(1.23–1.68)	(1.97–2.72)	(1.26–1.77)	(1.68–1.77)	(0.82–1.02)
**5–<10**	2.64	2.14	3.93	1.99	2.61	1.21
	(2.32–3.05)	(1.72–2.66)	(3.17–4.88)	(1.54–2.57)	(2.51–2.72)	(1.07–1.38)
**≥10**	3.35	2.24	5.66	2.92	3.22	1.49
	(2.38–4.71)	(1.30–3.86)	(3.32–9.66)	(1.33–6.44)	(2.86–3.63)	(1.16–1.92)

OR: odds ratio, CI: confidence interval.

^a^ Alzheimer’s disease dementia: patients with ICD-10 codes F00, and G30, without F01, F02, F03, F051, and G311.

^b^ Other cause dementia: patients with ICD-10 codes of F01, F02, F03, F051, G311, without F00 and G30.

^c^ Mixed dementia: patients with Alzheimer’s codes (F00 or G30) and any other cause dementia codes (F01, F02, F03, F051, G311) simultaneously.

^d^ The results were calculated by integrating ORs from results of all individual comorbidity status-based subgroups. Refer to S3 Table for the individual univariate ORs for each subgroup.

PIMs that exhibited significant interactions with polypharmacy were anticholinergics and H2-receptor antagonists (p<0.0001, S4 Table). Among comorbidities, hypertension, peripheral or cerebrovascular disease, congestive heart failure, hemiplegia, diabetes, depression, all other mental disorders, chronic obstructive pulmonary disease, peptic ulcer disease, and chronic liver disease showed significant interactions with polypharmacy (p<0.001, S4 Table).

We also compared ORs for dementia according to the interactions between comorbidities, PIMs, and polypharmacy. When not considering the interaction terms, the adjusted ORs increased significantly with the increasing levels of polypharmacy even as PIMs or the group of non-interacting diseases were included as variables. However, the magnitude of increase in ORs diminished when the group of interacting diseases was included in the analysis. When interaction terms were included in the analysis, the ORs for dementia in the polypharmacy group exhibited stronger increases in the absence of interacting PIMs or comorbidities but decreased and lost statistical significance when polypharmacy-interacting drugs or diseases were present (Table 4).

**Table 4:** Multivariable logistic regression analysis with or without interaction terms.

Variables	OR (95% CI) for dementia						
	Without the inclusion of interactions			With the inclusion of interactions (polypharmacy x interaction terms^b^^,^^d^)			
**<1**^a^	1	1	1	1	1	1	1
**1–<5**^a^	1.39	1.34	0.86	1.98	1.20	3.01	0.83
	(1.25–1.53)	(1.21–1.48)	(0.77–0.96)	(1.63–2.40)	(1.07–1.34)	(1.68–5.40)	(0.74–0.93)
**5–<10**^a^	1.92	1.81	1.17	2.58	1.70	- ^e^	1.15
	(1.67–2.21)	(1.57–2.08)	(1.01–1.36)	(1.75–3.81)	(1.46–1.99)		(0.99–1.34)
**≥10**^a^	2.23	2.11	1.43	- ^e^	2.10	- ^e^	1.40
	(1.57–3.18)	(1.48–3.01)	(1.00–2.05)		(1.47–3.00)		(0.97–2.01)
**Benzodiazepine (short/intermediate-acting)**	1.27	1.24	1.17	1.29	1.29	1.18	1.18
	(1.15–1.41)	(1.12–1.37)	(1.05–1.30)	(1.17–1.43)	(1.17–1.43)	(1.06–1.31)	(1.06–1.31)
**Benzodiazepine (long-acting)**	1.08	1.06	1.03	1.11	1.11	1.04	1.04
	(0.98–1.19)	(0.96–1.16)	(0.93–1.14)	(1.01–1.21)	(1.01–1.21)	(0.94–1.15)	(0.94–1.15)
**Benzodiazepine (receptor agonist)**	1.45	1.44	1.38	1.44	1.44	1.39	1.39
	(1.23–1.70)	(1.23–1.69)	(1.17–1.63)	(1.23–1.67)	(1.23–1.67)	(1.18–1.64)	(1.18–1.64)
**Anticholinergic drugs**^b^	1.29	1.28	1.09	absence	presence	1.09	1.09
	(1.18–1.42)	(1.17–1.41)	(0.99–1.21)			(0.98–1.20)	(0.98–1.20)
**H2-receptor antagonists**^b^	1.17	1.16	0.95	absence	presence	0.95	0.95
	(1.07–1.29)	(1.05–1.28)	(0.86–1.06)			(0.85–1.05)	(0.85–1.05)
**Group of non-interacting comorbidities**^c^	-	1.87	1.68	-	-	1.69	1.69
		(1.67–2.10)	(1.49–1.89)			(1.50–1.90)	(1.50–1.90)
**Group of interacting comorbidities**^d^	-	-	6.53	-	-	absence	presence
			(5.37–7.29)				

OR: odds ratio, CI: confidence interval.

^a^ the number of average prescribed daily drugs analyzed for the 2-year look-back period.

^b^ polypharmacy interacting drugs: anticholinergic drugs and H2-receptor antagonists.

^c^ group of comorbidities showing non-significant interaction: concomitant diseases which did not show significant interaction with polypharmacy.

^d^ polypharmacy interacting comorbidities: concomitant diseases that showed significant interactions such as hypertension, peripheral or cerebrovascular disease, congestive heart failure, hemiplegia, diabetes, depression, all other mental disorders, chronic obstructive pulmonary disease, peptic ulcer disease, and chronic liver disease.

^e^ not analyzed due to limited sample size (n<5).

## Discussion

We conducted a nested case-control study using a 12-year longitudinal cohort database representing the overall South Korean population, and our results confirm the association between polypharmacy and dementia. In a previous study, Lai *et al*. compared polypharmacy status among elderly people with and without dementia using the National Health Insurance data in Taiwan [20]. Their results showed that the risk of dementia increased with medication use, and confirmed that cerebrovascular disease interacted with polypharmacy. In comparison to the study by Lai *et al*., we excluded all hospitalized patients and only included outpatients to focus on the newly diagnosed dementia cases. Additionally, in our study, we matched the cases and controls for age and sex, which are major confounding factors for dementia. Furthermore, we used sophisticated analysis methods such as adjustment for PIM, and multiple logistic regression analyses that included interactions between polypharmacy and comorbidities or PIM to obtain more robust data on the association between polypharmacy and dementia.

A previous study by Draper *et al*. noted that approximately 25% dementia patients were hospitalized and that the average length of hospital stay for dementia patients was nearly double that of the non-dementia patients (16.5 days versus 8.9 days). They further reported that dementia was identified as the principal reason for hospitalization in only 6% of the cases [24]. In another study conducted in South Korea to assess the number of daily prescription drugs administered, the average number of daily prescribed drugs was 10% higher in the case group that included inpatients compared to the group that consisted only of outpatients [25]. Therefore, the exclusion of subjects with prior hospitalization history in this study is a conservative approach that lowers the possibility of including serious comorbidities among the newly diagnosed dementia cases.

We recorded a maximum of five comorbidities or CCI diseases for each patient, and these diseases are likely to be correlated to one another. Thus, a subgroup with a specific comorbidity could have other comorbidities simultaneously. Therefore, multicollinearity may be a concern when assessing comorbidities which have high correlation in one model. Various statistical methods have been recommended to overcome multicollinearity [26]. In this study, we used two approaches; firstly, we integrated the results from univariate logistic regression analyses conducted on patient subgroups with and without individual comorbidities. Secondly, based on their association with polypharmacy, we divided the comorbidities into interacting and non-interacting groups and conducted multiple logistic regression analyses to compare the results obtained by including and excluding the interaction terms. The association between polypharmacy and dementia remained statistically significant even after adjusting for comorbidities and PIM use even though the magnitude of the association decreased compared to the unadjusted values. It should be noted that the results of the association analysis depend on the statistical methods used.

There are several explanations for the association between polypharmacy and dementia. Polypharmacy may lead to a higher risk of dementia-related PIM use, and this may occur irrespective of whether or not the PIM is identified. The Beers criteria identify anticholinergic drugs, benzodiazepines, and H2-receptor antagonists as established PIMs for dementia due to their adverse effects on the central nervous system [23, 27–30]. As shown in Table 2, higher exposure to these PIMs was associated with higher risk of dementia. Thus, these PIMs should only be prescribed when essential, and prolonged use among the elderly should be avoided. In addition to the established PIMs listed in the Beers criteria, numerous other PIMs (such as proton pump inhibitors, antipsychotics, antidepressants, and opioids) may also be associated with dementia; however, the mechanisms for these associations are not yet clear [28, 31, 32]. Although it is generally known that established PIMs worsen dementia, there remain controversies on PIM use and the risk of developing dementia. A previous study reported a case of dementia that was reversed following the withdrawal of some prescribed drugs, and the authors emphasized the grave implications of polypharmacy on multiple comorbidities in dementia patients [33, 34]. Studies indicate that properly managed polypharmacy may help prevent, or delay, the development of dementia; however, further work is needed to verify this.

Additionally, the use of PIMs may cause continuous cognitive impairment. Dementia refers to a complex clinical syndrome that includes cognitive impairment and a decline in mental skills such as learning, language, and intelligence. This can be due to various causes and results in various functional impairments [35]. Wolkowitz *et al*. reported that following long-term steroid use, patients may develop prominent and persistent cognitive impairments due to drug-related neurotoxicity [36, 37]. Several experimental studies have been conducted to assess the impact of polypharmacy on cognitive function; however, the study results remain controversial. Jykka *et al*. assessed cognitive capacity using the Mini-Mental Status Examination (MMSE) scores, and compared them based on the polypharmacy levels. They noted that cases with excessive polypharmacy (≥10 drugs) exhibited significantly decreased cognitive function compared to non-polypharmacy cases (0–5 drugs) [38]. However, other studies reported a lack of association between polypharmacy and cognitive impairment in men [12, 39]. Wang *et al*. measured the cognitive capacity of hospitalized men aged ≥80 years using the MMSE, and also reported a lack of correlation between the scores and polypharmacy [12]. In both studies, polypharmacy level was analyzed with respect to cognitive test scores, and not dementia diagnosis. As MMSE is only one component of the assessments made to reach a diagnosis of dementia, the scores may not exactly correlate with the diagnosis, as noted by Brown *et al*. [40]. Moreover, constant exposure to polypharmacy can result in cognitive impairment, that may, in long-term, develop into dementia.

In addition to polypharmacy, comorbidities also exhibited a strong influence on dementia. The cases in our study had a higher prevalence of comorbidities than the controls even before a dementia diagnosis. A previous study has noted the association between hypertension and dementia [41]. Similarly, in this study, we observed a significantly higher prevalence of hypertension in the cases (67% versus 49% in the controls) during the look-back period. In the subgroups of patients with specific comorbidities, the crude OR for dementia decreased and lost statistical significance, and the lowest OR for dementia was observed in the subgroup of patients with concomitant cerebrovascular diseases (the OR for 5–<10 used drugs was 0.61 with 95% CI of 0.33–1.10, with the <1 drug used as reference) (S3 Table). In a multiple regression analysis, similarly, the adjusted OR for dementia decreased in the presence of comorbidity (Table 4). Therefore, in our study population, comorbidity was directly associated with dementia and was also the primary reason for an increase in the number of drugs prescribed. These results highlight the need for the careful management of comorbidities to prevent dementia.

In this study, as an exploratory analysis, we also assessed the impact of polypharmacy on the three dementia types. We observed that the diagnosis of non-Alzheimer’s dementia was most strongly affected by the level of polypharmacy. We defined non-Alzheimer’s dementia as a diagnosis with no Alzheimer-specific ICD-10 codes, and vascular disease dementia was considered to be the major subtype of non-Alzheimer’s dementia. Based on a nationwide survey in Korea, the proportion of non-Alzheimer dementia cases was 28.2%; our results exhibited slightly higher proportion. The proportion of vascular dementia cases was 82% of all the non-Alzheimer types, Lewy body dementia, Parkinson’s disease with dementia, frontotemporal dementia, and alcohol-related dementia accounted for only 2–3%; and not specified dementia accounted for 9% of all the non-Alzheimer types [42]. Kim *et al*. compared the comorbidity status of community-dwelling dementia patients based on the types of dementia and reported that the total comorbidity score was significantly higher in vascular disease dementia than in Alzheimer’s disease dementia (7.49±3.53 versus 5.46±3.13, p<0.001) [43]. As noted above, non-Alzheimer’s dementia includes various subgroups; however, we could not analyze the effect of polypharmacy levels on these subtypes because the records of subtypes were frequently missed in the database. Comorbidity could be the main risk factor for polypharmacy and dementia based on Kim’s studies; however, further analysis should be followed to determine how polypharmacy independently affects the development of a specific dementia subtype.

Our results demonstrate the relationship between polypharmacy and dementia, highlighting the need for multilateral efforts to reduce polypharmacy among the elderly. Several other reasons necessitate the control of polypharmacy. First, excessive polypharmacy results in a higher risk of PIM administration [19, 44–47]. A survey showed that about 50% of the community-dwelling patients with Alzheimer’s disease received PIMs, and such medications were even prescribed by Alzheimer specialists [48]. Second, polypharmacy may be related to functional decline [49], and a decreased quality of life among elderly dementia patients [50]. In a Japanese study based on elderly people with dementia who recently started receiving community-dwelling care, the quality of life and score of daily living activities were compared for 6 months between the reduced medication number (intervention) group and non-intervention group. Intervention group maintained their quality of life even as the medication number decreased during 6 months and showed slightly, however significantly, greater scores of daily living activities group compared to non-intervention group [50]. Third, polypharmacy could increase mortality risks in limited life expectancy groups. A study showed that polypharmacy was related with an increased mortality risk for the end-staged patients with advanced cognitive impairment in nursing homes; therefore, risks and benefits of polypharmacy should be evaluated, and the drug regimen should be improved to be as simple as possible for those patients [51]. Lastly, pill burden should be considered in addition to the risks associated with polypharmacy. We defined the level of polypharmacy based on the total number of either the type of drugs or the active ingredients in the drugs; therefore, the actual number of pills may be a few folds higher. Furthermore, it must be noted that some patients have difficulties in swallowing functions and taking multiple pills daily could affect adherence to the prescribed medication and treatment outcomes [52].

It may not be possible to completely avoid PIMs or exercise adequate caution in the administration of PIMs that negatively affect dementia as their mechanisms of action and impacts are diverse, and even undetermined in some cases. Therefore, practically, prescribing only the essential drugs would be the safest option for elderly dementia patients, and prescribing physicians should try to avoid increasing the number of medications [13, 53]. The need for deprescription has been recognized widely; however, policy measures to manage polypharmacy should be more actively implemented and executed with the increase in the elderly population and in the rising incidence of dementia. In addition, the causal relationship between polypharmacy and dementia, and the risks associated with the drugs used for treating dementia should be studied in the future.

This study is limited in its generalizability to other populations as all study subjects were Korean, and the database had limited information on patients’ clinical and laboratory data. For instance, previous studies have reported the association between body mass index (BMI) and the incidence of dementia [54, 55], and Koreans generally tend to have lower BMI than westerners. Based on the national health and nutrition examination surveys conducted in Korea and in the United States, the average BMI among the elderly population was 23.8 kg/m^2^ (SD: 3.2) in Korea [56], and 28.3 kg/m^2^ (SD: 7.1) in the United States [57]. Other limitations of this study are similar to those studies, which utilized reimbursement claim data for analysis [25]. The data on the number of drugs used is based solely on prescriptions, and the actual administration may be different. Moreover, over-the-counter, complementary, and alternative medications were not included in the list of administered/prescribed drugs. Additionally, PIM exposure did not include information on the level of drug use, such as the dosage of the administered drugs. Our study utilized information from insurance claims database, and, therefore, we could not access the patients’ medical records. We classified cases based on the ICD-10 codes included in the database, and although the diagnoses were recorded by health care professionals, there remains the possibility of misclassification or misdiagnosis. Polypharmacy showed a stronger association with vascular dementia than with Alzheimer’s disease dementia; however, the risk of misdiagnosis may be larger for the subtypes of dementia. Our evaluation of the association between polypharmacy and individual dementia subtypes were thus limited, and future studies should elucidate these associations further, utilizing well-defined case selection and study design.

This study has several strengths. The use of multiple statistical approaches and the utilization of long-term cohort data from real world setting, representative of the entire South Korean population provides robustness to our results and strongly emphasizes the association between polypharmacy and dementia.

In conclusion, polypharmacy was significantly associated with the incidence of dementia even after the association was adjusted for dementia-related PIM use. Additional efforts are needed to maintain or reduce the number of prescribed drugs in order to reduce the risk of dementia.

## Supporting Information

S1 Table The ICD-10 codes used for analysis. (DOCX)

S2 Table Demographics and clinical information for the dementia type-based subgroups. SD: standard deviation, CCI: Charlson comorbidity index.
^a^ Alzheimer’s disease dementia: patients with ICD-10 codes F00 and G30, without F01, F02, F03, F051, and G311.
^b^ Other cause dementia: patients with ICD-10 codes F01, F02, F03, F051, and G311, without F00 and G30.
^c^ Mixed dementia: patients with Alzheimer’s codes (F00 or G30) and any other cause dementia codes (F01, F02, F03, F051, and G311) simultaneously.
^d^ D1: myocardial infarction, D2: congestive heart failure, D3: peripheral vascular disease, D4: cerebrovascular vascular disease, D5: dementia, D6: chronic obstructive pulmonary disease, D7: connective tissue disease, D8: peptic ulcer disease, D9: chronic liver disease, D10: diabetes mellitus (uncomplicated), D11: diabetes mellitus (complicated), D12: Hemiplegia, D13: moderate/severe kidney disease, D14: tumor, leukemia, lymphoma, D15: moderate/severe liver disease, D16: metastatic solid tumor, D17: acquired immune deficiency syndrome, D18: hypertension, D19: depression, D20: delirium, D21: behavioral disorders due to alcohol, D22: schizophrenia/psychotic disorders, D23: all other mental disorders.
(DOCX)

S3 Table The results of univariate logistic regression analyses according to patient comorbidity subgroup. OR: odds ratio, CI: confidence interval, PIM: potentially inappropriate medication, NA: not applicable.
^a^ Data not available due to the small size for matched controls.
(DOCX)

S4 Table Interaction between polypharmacy and predictor variables. (DOCX)

## References

[1] National Institute on Aging / National Institute of Health. Gloabal health and aging. World Health Organization, 2011 NIH Publication no. 11–7737.

[2] Korean Statisticcal Information Service. Estimated future population.http://kosis.kr/statHtml/statHtml.do?orgId=101&tblId=DT_1B01B01&vw_cd=&list_id=&scrId=&seqNo=&lang_mode=ko&obj_var_id=&itm_id=&conn_path=K1&path=.

[3] YH Jung. A study on the effective chronic disease management. Korea Institute for Health and Social Affairs: Korea Institute for Health and Social Affairs, 2013 Contract No.: 2013-31-19.

[4] Martin Prince EA, Maëlenn Guerchet, Matthew Prina,. World Alzheimer Report 2014; Dementia and Risk Reduction- An analysis of protecive and modifiable factors. Alzheimer’s Disease International (ADI), London, 2014.

[5] Peng D. The global burden of demential worldwide. January 13–14, 2011, Geneva, Switzerland IAGG/WHO/SFGG Workshop Health promotion program on prevention of late onset dementia: 2011.

[6] World Health Organization. Ageing and Health Technical Report Vol.5: A Glossary of Terms for Community Health Care and Services for Older

[7] Gillette C, Prunty L, Wolcott J, Broedel-Zaugg K. A new lexicon for polypharmacy: Implications for research, practice, and education. Research in social & administrative pharmacy: RSAP. 2015;11(3):468–71. 25280463 10.1016/j.sapharm.2014.08.010

[8] Turner JP, Jamsen KM, Shakib S, Singhal N, Prowse R, Bell JS. Polypharmacy cut-points in older people with cancer: how many medications are too many? Supportive care in cancer: official journal of the Multinational Association of Supportive Care in Cancer. 2015. 10.1007/s00520-015-2970-8 26449548

[9] Frazier SC. Health outcomes and polypharmacy in elderly individuals: an integrated literature review. Journal of gerontological nursing. 2005;31(9):4–11. 16190007 10.3928/0098-9134-20050901-04

[10] Franchi C, Marcucci M, Mannucci PM, Tettamanti M, Pasina L, Fortino I, et al Changes in clinical outcomes for community-dwelling older people exposed to incident chronic polypharmacy: a comparison between 2001 and 2009. Pharmacoepidemiology and drug safety. 2016;25(2):204–11. 10.1002/pds.3938 26687829

[11] Fried TR, O'Leary J, Towle V, Goldstein MK, Trentalange M, Martin DK. Health outcomes associated with polypharmacy in community-dwelling older adults: a systematic review. Journal of the American Geriatrics Society. 2014;62(12):2261–72. 10.1111/jgs.13153 25516023 PMC4270076

[12] Wang R, Chen L, Fan L, Gao D, Liang Z, He J, et al Incidence and Effects of Polypharmacy on Clinical Outcome among Patients Aged 80+: A Five-Year Follow-Up Study. PloS one. 2015;10(11):e0142123 10.1371/journal.pone.0142123 26554710 PMC4640711

[13] Garfinkel D, Ilhan B, Bahat G. Routine deprescribing of chronic medications to combat polypharmacy. Therapeutic advances in drug safety. 2015;6(6):212–33. 10.1177/2042098615613984 26668713 PMC4667766

[14] Marengoni A, Onder G. Guidelines, polypharmacy, and drug-drug interactions in patients with multimorbidity. Bmj. 2015;350:h1059 10.1136/bmj.h1059 25761379

[15] Fried TR, Tinetti ME, Iannone L, O'Leary JR, Towle V, Van Ness PH. Health outcome prioritization as a tool for decision making among older persons with multiple chronic conditions. Archives of internal medicine. 2011;171(20):1854–6. 10.1001/archinternmed.2011.424 21949032 PMC4036681

[16] Patterson SM, Cadogan CA, Kerse N, Cardwell CR, Bradley MC, Ryan C, et al Interventions to improve the appropriate use of polypharmacy for older people. The Cochrane database of systematic reviews. 2014;10:CD008165. 10.1002/14651858.CD008165.pub3 25288041

[17] Hajjar ER, Hanlon JT, Artz MB, Lindblad CI, Pieper CF, Sloane RJ, et al Adverse drug reaction risk factors in older outpatients. The American journal of geriatric pharmacotherapy. 2003;1(2):82–9. 15555470 10.1016/s1543-5946(03)90004-3

[18] Gallacher KI, Batty GD, McLean G, Mercer SW, Guthrie B, May CR, et al Stroke, multimorbidity and polypharmacy in a nationally representative sample of 1,424,378 patients in Scotland: implications for treatment burden. BMC medicine. 2014;12:151 10.1186/s12916-014-0151-0 25280748 PMC4220053

[19] Lau DT, Mercaldo ND, Harris AT, Trittschuh E, Shega J, Weintraub S. Polypharmacy and potentially inappropriate medication use among community-dwelling elders with dementia. Alzheimer disease and associated disorders. 2010;24(1):56–63. 10.1097/WAD.0b013e31819d6ec9 19561441 PMC2837122

[20] Lai SW, Lin CH, Liao KF, Su LT, Sung FC, Lin CC. Association between polypharmacy and dementia in older people: a population-based case-control study in Taiwan. Geriatrics & gerontology international. 2012;12(3):491–8. 22233227 10.1111/j.1447-0594.2011.00800.x

[21] Lee J, Lee JS, Park SH, Shin SA, Kim K. Cohort Profile: The National Health Insurance Service-National Sample Cohort (NHIS-NSC), South Korea. International journal of epidemiology. 2016. 10.1093/ije/dyv319 26822938

[22] Quan H, Sundararajan V, Halfon P, Fong A, Burnand B, Luthi JC, et al Coding algorithms for defining comorbidities in ICD-9-CM and ICD-10 administrative data. Medical care. 2005;43(11):1130–9. 16224307 10.1097/01.mlr.0000182534.19832.83

[23] By the American Geriatrics Society Beers Criteria Update Expert P. American Geriatrics Society 2015 Updated Beers Criteria for Potentially Inappropriate Medication Use in Older Adults. Journal of the American Geriatrics Society. 2015;63(11):2227–46. 10.1111/jgs.13702 26446832

[24] Draper B, Karmel R, Gibson D, Peut A, Anderson P. The Hospital Dementia Services Project: age differences in hospital stays for older people with and without dementia. International psychogeriatrics. 2011;23(10):1649–58. Epub 2011/09/10. 10.1017/S1041610211001694 21902861

[25] Park HY, Ryu HN, Shim MK, Sohn HS, Kwon JW. Prescribed drugs and polypharmacy in healthcare service users in South Korea: an analysis based on National Health Insurance Claims data. International journal of clinical pharmacology and therapeutics. 2016;54(5):369–77. Epub 2016/03/24. 10.5414/CP202484 27007996

[26] Dohoo IR, Ducrot C, Fourichon C, Donald A, Hurnik D. An overview of techniques for dealing with large numbers of independent variables in epidemiologic studies. Preventive veterinary medicine. 1997;29(3):221–39. Epub 1997/01/01. 9234406 10.1016/s0167-5877(96)01074-4

[27] Gray SL, Anderson ML, Dublin S, Hanlon JT, Hubbard R, Walker R, et al Cumulative use of strong anticholinergics and incident dementia: a prospective cohort study. JAMA internal medicine. 2015;175(3):401–7. Epub 2015/01/27. 10.1001/jamainternmed.2014.7663 25621434 PMC4358759

[28] Lebedev AV, Beyer MK, Fritze F, Westman E, Ballard C, Aarsland D. Cortical changes associated with depression and antidepressant use in Alzheimer and Lewy body dementia: an MRI surface-based morphometric study. The American journal of geriatric psychiatry: official journal of the American Association for Geriatric Psychiatry. 2014;22(1):4–13 e1. 23880336 10.1016/j.jagp.2013.02.004

[29] Zhong G, Wang Y, Zhang Y, Zhao Y. Association between Benzodiazepine Use and Dementia: A Meta-Analysis. PloS one. 2015;10(5):e0127836 Epub 2015/05/29. 10.1371/journal.pone.0127836 26016483 PMC4446315

[30] Boustani M, Hall KS, Lane KA, Aljadhey H, Gao S, Unverzagt F, et al The association between cognition and histamine-2 receptor antagonists in African Americans. Journal of the American Geriatrics Society. 2007;55(8):1248–53. Epub 2007/07/31. 10.1111/j.1532-5415.2007.01270.x 17661965 PMC2860609

[31] Haenisch B, von Holt K, Wiese B, Prokein J, Lange C, Ernst A, et al Risk of dementia in elderly patients with the use of proton pump inhibitors. European archives of psychiatry and clinical neuroscience. 2015;265(5):419–28. 10.1007/s00406-014-0554-0 25341874

[32] Dublin S, Walker RL, Gray SL, Hubbard RA, Anderson ML, Yu O, et al Prescription Opioids and Risk of Dementia or Cognitive Decline: A Prospective Cohort Study. Journal of the American Geriatrics Society. 2015;63(8):1519–26. 10.1111/jgs.13562 26289681 PMC4776316

[33] Gupta M, Singh R, Singh K, Lehl SS. Reversible dementia and gait disturbance as a result of polypharmacy. BMJ case reports. 2013;2013. Epub 2013/03/28. 10.1136/bcr-2013-008932 PMC3618831 23531935

[34] Evans MD, Shinar R, Yaari R. Reversible dementia and gait disturbance after prolonged use of valproic acid. Seizure. 2011;20(6):509–11. Epub 2011/03/26. 10.1016/j.seizure.2011.02.009 21435910

[35] LoGiudice D, Watson R. Dementia in older people: an update. Internal medicine journal. 2014;44(11):1066–73. 10.1111/imj.12572 25367725

[36] Wolkowitz OM, Lupien SJ, Bigler E, Levin RB, Canick J. The "steroid dementia syndrome": an unrecognized complication of glucocorticoid treatment. Annals of the New York Academy of Sciences. 2004;1032:191–4. Epub 2005/01/29. 10.1196/annals.1314.018 15677408

[37] Wolkowitz OM, Lupien SJ, Bigler ED. The "steroid dementia syndrome": a possible model of human glucocorticoid neurotoxicity. Neurocase. 2007;13(3):189–200. Epub 2007/09/06. 10.1080/13554790701475468 17786779

[38] Jyrkka J, Enlund H, Lavikainen P, Sulkava R, Hartikainen S. Association of polypharmacy with nutritional status, functional ability and cognitive capacity over a three-year period in an elderly population. Pharmacoepidemiology and drug safety. 2011;20(5):514–22. 10.1002/pds.2116 21308855

[39] Gnjidic D, Hilmer SN, Blyth FM, Naganathan V, Waite L, Seibel MJ, et al Polypharmacy cutoff and outcomes: five or more medicines were used to identify community-dwelling older men at risk of different adverse outcomes. Journal of clinical epidemiology. 2012;65(9):989–95. 10.1016/j.jclinepi.2012.02.018 22742913

[40] Brown J. The use and misuse of short cognitive tests in the diagnosis of dementia. Journal of neurology, neurosurgery, and psychiatry. 2015;86(6):680–5. Epub 2014/11/21. 10.1136/jnnp-2014-309086 25411547

[41] Perrotta M, Lembo G, Carnevale D. Hypertension and Dementia: Epidemiological and Experimental Evidence Revealing a Detrimental Relationship. International journal of molecular sciences. 2016;17(3). Epub 2016/03/24. 10.3390/ijms17030347 PMC4813208 27005613

[42] Kim KW, Park JH, Kim MH, Kim MD, Kim BJ, Kim SK, et al A nationwide survey on the prevalence of dementia and mild cognitive impairment in South Korea. Journal of Alzheimer's disease: JAD. 2011;23(2):281–91. Epub 2010/12/24. 10.3233/JAD-2010-101221 21178286

[43] KW Kim. A study on the Demented Elderly Seoul National University Bundang Hospital; 2011. http://www.google.co.kr/url?url=http://www.prism.go.kr/homepage/researchCommon/downloadResearchAttachFile.do%3Bjsessionid%DCD0D96D5D3BD1B1123E20C698BC923E.node02%3Fwork_key%3D001%26file_type%3DCPR%26seq_no%3D001%26pdf_conv_yn%3DY%26research_id%3D1351000-201000126&rct=j&frm=1&q=&esrc=s&sa=U&ved=0ahUKEwiSpIvx4KPNAhUBHJQKHZp_DgUQFgglMAM&sig2=5sGD5Je42mvet4Pjahim9Q&usg=AFQjCNG_iMoLrKmwR3iUpK6JFI-EqFTK1Q.

[44] Viswanathan H, Bharmal M, Thomas J 3rd. Prevalence and correlates of potentially inappropriate prescribing among ambulatory older patients in the year 2001: comparison of three explicit criteria. Clinical therapeutics. 2005;27(1):88–99. Epub 2005/03/15. 10.1016/j.clinthera.2005.01.009 15763610

[45] Hwang HJ, Kim SH, Lee KS. Potentially Inappropriate Medications in the Elderly in Korean Long-Term Care Facilities. Drugs—real world outcomes. 2015;2(4):355–61. Epub 2015/12/23. 10.1007/s40801-015-0046-1 26689669 PMC4674516

[46] Alhmoud E, Khalifa S, Bahi AA. Prevalence and predictors of potentially inappropriate medications among home care elderly patients in Qatar. International journal of clinical pharmacy. 2015;37(5):815–21. 10.1007/s11096-015-0125-0 25986290

[47] Andersen F, Viitanen M, Halvorsen DS, Straume B, Engstad TA. Co-morbidity and drug treatment in Alzheimer's disease. A cross sectional study of participants in the dementia study in northern Norway. BMC geriatrics. 2011;11:58 Epub 2011/10/06. 10.1186/1471-2318-11-58 21970467 PMC3204237

[48] Montastruc F, Gardette V, Cantet C, Piau A, Lapeyre-Mestre M, Vellas B, et al Potentially inappropriate medication use among patients with Alzheimer disease in the REAL.FR cohort: be aware of atropinic and benzodiazepine drugs! European journal of clinical pharmacology. 2013;69(8):1589–97. Epub 2013/04/17. 10.1007/s00228-013-1506-8 23588564

[49] Lau DT, Mercaldo ND, Shega JW, Rademaker A, Weintraub S. Functional decline associated with polypharmacy and potentially inappropriate medications in community-dwelling older adults with dementia. American journal of Alzheimer's disease and other dementias. 2011;26(8):606–15. 10.1177/1533317511432734 22207646 PMC3298080

[50] Sakakibara M, Igarashi A, Takase Y, Kamei H, Nabeshima T. Effects of Prescription Drug Reduction on Quality of Life in Community-Dwelling Patients with Dementia. Journal of pharmacy & pharmaceutical sciences: a publication of the Canadian Society for Pharmaceutical Sciences, Societe canadienne des sciences pharmaceutiques. 2015;18(5):705–12. Epub 2015/12/17. 10.18433/j37p5x 26670367

[51] Onder G, Liperoti R, Foebel A, Fialova D, Topinkova E, van der Roest HG, et al Polypharmacy and mortality among nursing home residents with advanced cognitive impairment: results from the SHELTER study. Journal of the American Medical Directors Association. 2013;14(6):450.e7–12. Epub 2013/05/08. 10.1016/j.jamda.2013.03.014 23647778

[52] Fields J, Go JT, Schulze KS. Pill Properties that Cause Dysphagia and Treatment Failure. Current therapeutic research, clinical and experimental. 2015;77:79–82. Epub 2015/11/07. 10.1016/j.curtheres.2015.08.002 26543509 PMC4589822

[53] Scott IA, Hilmer SN, Reeve E, Potter K, Le Couteur D, Rigby D, et al Reducing inappropriate polypharmacy: the process of deprescribing. JAMA internal medicine. 2015;175(5):827–34. 10.1001/jamainternmed.2015.0324 25798731

[54] Garcia-Ptacek S, Faxen-Irving G, Cermakova P, Eriksdotter M, Religa D. Body mass index in dementia. European journal of clinical nutrition. 2014;68(11):1204–9. Epub 2014/10/02. 10.1038/ejcn.2014.199 25271014

[55] Pedditizi E, Peters R, Beckett N. The risk of overweight/obesity in mid-life and late life for the development of dementia: a systematic review and meta-analysis of longitudinal studies. Age and ageing. 2016;45(1):14–21. Epub 2016/01/15. 10.1093/ageing/afv151 26764391

[56] Kim KN, Y J. Predictors of Health-related Quality of Life in Korean Elderly: Based on the 2013 Korea National Health and Nutrition Examination Survey. Asia-pacific Journal of Multimedia Services Convergent with Art, Humanities, and Sociology. 2015;5:197–205.

[57] Grandner MA, Schopfer EA, Sands-Lincoln M, Jackson N, Malhotra A. Relationship between sleep duration and body mass index depends on age. Obesity (Silver Spring, Md). 2015;23(12):2491–8. Epub 2016/01/05. 10.1002/oby.21247 PMC4700549 26727118

