# Supplementary material for: The Association between Polypharmacy and Dementia: A Nested Case-Control Study Based on a 12-Year Longitudinal Cohort Database in South Korea
Source: PLoS One. 2017 Jan 5;12(1):e0169463. doi: 10.1371/journal.pone.0169463 (PMC5215897; doi:10.1371/journal.pone.0169463)
Supplement: S1 Table — (DOCX) [file pone.0169463.s001.docx]

**S1 Table. The ICD-10 codes used for analysis**

| Disease | | ICD-10 code |
| --- | --- | --- |
| Dementia | | F00, F01, F02, F03, F051, G30, G311 |
| CCI  diseases | Myocardial infarction | I21, I122, I1252 |
|  | Congestive heart failure | I43, I50, I099, I110, I130, I132, I255, I420, I425, I426, I427, I428, I429, P290 |
|  | Peripheral vascular disease | I70, I71, I731, I738, I739, I771, I790, I792, K551, K558, K559, Z958, Z959 |
|  | Dementia | Same as the above-mentioned codes for dementia |
|  | Cerebrovascular disease | G45, G46, I60, I61, I62, I63, I64, I65, I66, I67, I68, I69, H340 |
|  | Chronic lung disease | J40, J41, J42, J43, J44, J45, J46, J47, J60, J61, J62, J63, J64, J65, J66, J67, I278, I279, J684, J701, J703. |
|  | Connective tissue disease | M05, M06, M32, M33, M34, M315, M351, M353, M360 |
|  | Peptic ulcer | K25, K26, K27, K28 |
|  | Chronic liver disease | B18, K73, K74, K700, K701, K702, K703, K709, K713, K714, K715, K717, K760, K762, K763, K764, K768, K769, Z944 |
|  | Diabetes mellitus (uncomplicated) | E100, E101, E106, E108, E109, E110, E111, E116, E118, E119, E120, E121, E126, E128, E129, E130, E131, E136, E138, E139, E140, E141, E146, E148, E149 |
|  | Diabetes mellitus (complicated) | E102, E103, E104, E105, E107, E112, E113, E114, E115, E117, E122, E123, E124, E125, E127, E132, E133, E134, E135, E137, E142, E143, E144, E145, E147 |
|  | Hemiplegia | G81, G82, G041, G114, G801, G802, G830, G831, G832, G833, G834, G839 |
|  | Moderate/severe kidney disease | N18, N19, I120, I131, N32, N33, N34, N35, N36, N37, N52, N53, N54, N55, N56, N57, N250, Z490, Z491, Z492, Z940, Z992 |
|  | Tumor, leukemia, lymphoma | C00, C01, C02, C03, C04, C05, C06, C07, C08, C09, C10, C11, C12, C13, C14, C15, C16, C17, C18, C19, C20, C21, C22, C23, C24, C25, C26, C30, C31, C32, C33, C34, C37, C38, C39, C40, C41, C43, C45, C46, C47, C48, C60, C61, C62, C63, C64, C65, C66, C67, C68, C69, C70, C71, C72, C73, C74, C75, C76, C81, C82, C83, C84, C85, C88, C90, C91, C92, C93, C94, C95, C96, C97 |
|  | Moderate/severe liver disease | I850, I859, I864, I982, K704, K711, K721, K729, K765, K766, K767 |
|  | Metastatic solid tumor | C77, C78, C79, C80 |
|  | Acquired immune deficiency syndrome | B20, B21, B22, B24 |
| Hypertension | | I10–15 |
| Depression | | F32, F33 |
| Delirium | | F05 (excluding F051) |
| Behavioral disorders due to alcohol | | F10 |
| Schizophrenia/ psychotic disorders | | F20–29 |
| All other mental disorders | | All other F codes excluding hypertension, depression, delirium, behavioral disorders due to alcohol, schizophrenia, and psychotic disorders. |
