# Supplementary material for: The Association between Polypharmacy and Dementia: A Nested Case-Control Study Based on a 12-Year Longitudinal Cohort Database in South Korea
Source: PLoS One. 2017 Jan 5;12(1):e0169463. doi: 10.1371/journal.pone.0169463 (PMC5215897; doi:10.1371/journal.pone.0169463)
Supplement: S2 Table — SD: standard deviation, CCI: Charlson comorbidity index. a Alzheimer’s disease dementia: patients with ICD-10 codes F00 and G30, without F01, F02, F03, F051, and G311. b Other cause dementia: patients with ICD-10 codes F01, F02, F03, F051, and G311, without F00 and G30. c Mixed dementia: patients with Alzheimer’s codes (F00 or G30) and any other cause dementia codes (F01, F02, F03, F051, and G311) simultaneously. d D1: myocardial infarction, D2: congestive heart failure, D3: peripheral vascular disease, D4: cerebrovascular vascular disease, D5: dementia, D6: chronic obstructive pulmonary disease, D7: connective tissue disease, D8: peptic ulcer disease, D9: chronic liver disease, D10: diabetes mellitus (uncomplicated), D11: diabetes mellitus (complicated), D12: Hemiplegia, D13: moderate/severe kidney disease, D14: tumor, leukemia, lymphoma, D15: moderate/severe liver disease, D16: metastatic solid tumor, D17: acquired immune deficiency syndrome, D18: hypertension, D19: depression, D20: delirium, D21: behavioral disorders due to alcohol, D22: schizophrenia/psychotic disorders, D23: all other mental disorders. (DOCX) [file pone.0169463.s002.docx]

**S2 Table. Demographics and clinical information for the dementia type-based subgroups**

| Type of dementia | Alzheimer’s disease dementia^a^ | | Other cause dementia^b^ | | Mixed dementia^c^ | |
| --- | --- | --- | --- | --- | --- | --- |
|  | Cases | Controls | Cases | Controls | Cases | Controls |
|  | (n=1,841) | (n=1,841) | (n=2,139) | (n=2,139) | (n=1,582) | (n=1,582) |
|  | n (%) | n (%) | n (%) | n (%) | n (%) | n (%) |
| Male | 493 (27) | 493 (27) | 636 (30) | 636 (30) | 437 (28) | 437 (28) |
| Age Mean [SD] | 14.7 [1.4] | 14.7 [1.4] | 14.5 [1.4] | 14.5 [1.4] | 14.8 [1.3] | 14.8 [1.3] |
| 65–<75 years | 904 (49) | 904 (49) | 1,206 (56) | 1,256 (56) | 702 (44) | 702 (44) |
| ≥75 years | 937 (51) | 937 (51) | 933 (44) | 933 (44) | 880 (56) | 880 (56) |
| CCI score Mean [SD] | 3.6 [2.3] | 1.6 [2.0] | 3.8 [2.2] | 1.5 [1.9] | 3.5 [2.1] | 1.4 [1.7] |
| CCI diseases^d^ |  |  |  |  |  |  |
| D1 | 35 (2) | 25 (1) | 48 (2) | 20 (1) | 24 (2) | 13 (1) |
| D2 | 197 (11) | 126 (7) | 224 (10) | 115 (5) | 151 (10) | 81 (5) |
| D3 | 458 (25) | 294 (16) | 662 (31) | 319 (15) | 372 (24) | 218 (14) |
| D4 | 628 (34) | 224 (12) | 824 (39) | 218 (10) | 658 (42) | 149 (9) |
| D5 | 1,841 (100) | 0 (0) | 2,139 (100) | 0 (0) | 1,582 (100) | 0 (0) |
| D6 | 663 (36) | 518 (28) | 854 (40) | 594 (28) | 508 (32) | 388 (25) |
| D7 | 113 (6) | 59 (3) | 144 (7) | 67 (3) | 94 (6) | 58 (4) |
| D8 | 614 (33) | 436 (24) | 791 (37) | 505 (24) | 458 (29) | 331 (21) |
| D9 | 436 (24) | 271 (15) | 516 (24) | 320 (15) | 322 (20) | 201 (13) |
| D10 | 515 (28) | 341 (19) | 634 (30) | 386 (18) | 410 (26) | 251 (16) |
| D11 | 215 (12) | 148 (8) | 260 (12) | 144 (7) | 189 (12) | 102 (6) |
| D12 | 55 (3) | 18 (1) | 119 (6) | 18 (1) | 60 (4) | 7 (0) |
| D13 | 32 (2) | 22 (1) | 32 (1) | 23 (1) | 30 (2) | 13 (1) |
| D14 | 170 (9) | 17 (6) | 213 (10) | 124 (6) | 139 (9) | 78 (5) |
| D15 | 8 (0) | 7 (0) | 11 (1) | 3 (0) | 8 (1) | 2 (0) |
| D16 | 20 (1) | 8 (0) | 16 (1) | 15 (1) | 10 (1) | 9 (1) |
| D17 | 1 (0) | 0 (0) | 0 (0) | 0 (0) | 0 (0) | 0 (0) |
| Other comorbidities^d^ |  |  |  |  |  |  |
| D18 | 1,194 (65) | 922 (50) | 1,497 (70) | 1,036 (48) | 1,011 (64) | 744 (47) |
| D19 | 341 (19) | 107 (6) | 329 (15) | 147 (7) | 289 (18) | 72 (5) |
| D20 | 28 (2) | 2 (0) | 19 (1) | 1 (0) | 20 (1) | 1 (0) |
| D21 | 14 (1) | 2 (0) | 12 (1) | 7 (0) | 17 (1) | 2 (0) |
| D22 | 37 (2) | 3 (0) | 27 (1) | 2 (0) | 44 (3) | 3 (0) |
| D23 | 831 (45) | 433 (24) | 1014 (47) | 528 (25) | 655 (41) | 348 (22) |

SD: standard deviation, CCI: Charlson comorbidity index.

^a^ Alzheimer’s disease dementia: patients with ICD-10 codes F00 and G30, without F01, F02, F03, F051, and G311.

^b^ Other cause dementia: patients with ICD-10 codes F01, F02, F03, F051, and G311, without F00 and G30.

^c^ Mixed dementia: patients with Alzheimer’s codes (F00 or G30) and any other cause dementia codes (F01, F02, F03, F051, and G311) simultaneously.

^d^ D1: myocardial infarction, D2: congestive heart failure, D3: peripheral vascular disease, D4: cerebrovascular vascular disease, D5: dementia, D6: chronic obstructive pulmonary disease, D7: connective tissue disease, D8: peptic ulcer disease, D9: chronic liver disease, D10: diabetes mellitus (uncomplicated), D11: diabetes mellitus (complicated), D12: Hemiplegia, D13: moderate/severe kidney disease, D14: tumor, leukemia, lymphoma, D15: moderate/severe liver disease, D16: metastatic solid tumor, D17: acquired immune deficiency syndrome, D18: hypertension, D19: depression, D20: delirium, D21: behavioral disorders due to alcohol, D22: schizophrenia/psychotic disorders, D23: all other mental disorders.
