# Supplementary material for: The Association between Polypharmacy and Dementia: A Nested Case-Control Study Based on a 12-Year Longitudinal Cohort Database in South Korea
Source: PLoS One. 2017 Jan 5;12(1):e0169463. doi: 10.1371/journal.pone.0169463 (PMC5215897; doi:10.1371/journal.pone.0169463)
Supplement: S3 Table — OR: odds ratio, CI: confidence interval, PIM: potentially inappropriate medication, NA: not applicable. a Data not available due to the small size for matched controls. (DOCX) [file pone.0169463.s003.docx]

**S3 Table. The results of univariate logistic regression analyses according to patient comorbidity subgroup**

| Comorbidity | Poly-pharmacy level | Patient subgroup  with no specific comorbidities | | | Patient subgroup  with specific comorbidities | | |
| --- | --- | --- | --- | --- | --- | --- | --- |
|  |  | Matched n | Univariate OR (95% CI) | | Matched n | Univariate OR (95% CI) | |
|  |  |  | Without PIM adjustment | With PIM adjustment |  | Without PIM adjustment | With PIM adjustment |
| Depression, delirium, schizophrenia/  psychotic disorders, all other mental disorders | <1  1–<5  5–<10  ≥10 | 2,039 | 1  2.0 (1.7–2.4)  2.4 (1.8–3.1)  2.3 (0.9–5.8) | 1  1.7 (1.4–2.0)  2.0 (1.5–2.6)  1.5 (0.6–3.9) | 813 | 1  0.9 (0.7–1.2)  1.3 (1.0–1.8)  1.4 (0.7–2.7) | 1  1.0 (0.7–1.2)  1.5 (1.1–2.0)  1.6 (0.8–3.2) |
| Myocardial infarction | <1  1–<5  5–<10  ≥10 | 5,398 | 1  1.7 (1.5–1.9)  2.6 (2.3–3.0)  3.2 (2.3–4.6) | 1  1.4 (1.2–1.5)  1.9 (1.6–2.2)  2.1 (1.5–3.1) | 0 | NA | NA |
| Congestive heart failure | <1  1–<5  5–<10  ≥10 | 4,711 | 1  1.7 (1.6–1.9)  2.6 (2.3–3.0)  3.2 (2.1–4.8) | 1  1.4 (1.2–1.5)  1.9 (1.7–2.3)  2.1 (1.4–3.2) | 43 | 1  1.5 (0.4–6.3)  1.4 (0.3–6.1)  0.3 (0.0–3.8) | 1  2.6 (0.4–16.8)  0.5 (0.1–3.7)  0.3 (0.0–6.4) |
| Peripheral vascular disease | <1  1–<5  5–<10  ≥10 | 3,497 | 1  1.7 (1.5–1.9)  2.5 (2.1–3.0)  2.7 (1.6–4.4) | 1  1.3 (1.2–1.5)  1.8 (1.5–2.2)  1.8 (1.1–2.9) | 258 | 1  1.4 (0.9–2.4)  1.5 (0.8–2.8)  1.8 (0.6–5.2) | 1  1.3 (0.7–2.2)  1.3 (0.7–2.5)  1.6 (0.5–4.7) |
| Cerebrovascular disease | <1  1–<5  5–<10  ≥10 | 3,101 | 1  1.6 (1.4–1.8)  2.4 (2.0–3.0)  3.8 (2.0–7.4) | 1  1.3 (1.1–1.4)  1.8 (1.5–2.2)  2.5 (1.2–4.9) | 240 | 1  0.8 (0.5–1.3)  0.6 (0.3–1.1)  1.6 (0.4–5.6) | 1  0.7 (0.4–1.2)  0.5 (0.2–0.9)  1.1 (0.3–4.3) |
| Chronic obstructive pulmonary disease | <1  1–<5  5–<10  ≥10 | 2,635 | 1  1.8 (1.6–2.1)  2.5 (2.0–3.1)  3.1 (1.6–5.9) | 1  1.6 (1.3–1.8)  2.0 (1.6–2.5)  2.2 (1.1–4.3) | 598 | 1  0.9 (0.7–1.2)  1.5 (1.0–2.1)  2.3 (1.2–4.5) | 1  0.8 (0.6–1.0)  1.2 (0.8–1.7)  1.6 (0.8–3.3) |
| Connective tissue disease | <1  1–<5  5–<10  ≥10 | 5,052 | 1  1.7 (1.6–1.9)  2.7 (2.3–3.1)  3.9 (2.6–5.7) | 1  1.4 (1.3–1.6)  2.0 (1.7–2.3)  2.5 (1.7–3.8) | 25 | -^a^ | -^a^ |
| Peptic ulcer disease | <1  1–<5  5–<10  ≥10 | 2,903 | 1  1.8 (1.6–2.1)  2.7 (2.2–3.3)  2.8 (1.5–5.5) | 1  1.5 (1.3–1.8)  2.0 (1.6–2.5)  1.9 (1.0–3.8) | 476 | 1  1.0 (0.7–1.4)  1.2 (0.8–1.7)  1.7 (0.8–3.6) | 1  1.0 (0.7–1.4)  1.1 (0.8–1.7)  1.6 (0.7–3.4) |
| Chronic liver disease | <1  1–<5  5–<10  ≥10 | 3,696 | 1  1.7 (1.6–2.0)  2.7 (2.3–3.2)  3.5 (2.1–5.8) | 1  1.4 (1.3–1.6)  2.0 (1.7–2.4)  2.4 (1.4–4.0) | 200 | 1  1.0 (0.6–1.7)  1.3 (0.7–2.4)  1.1 (0.4–3.2) | 1  1.0 (0.6–1.7)  1.2 (0.6–2.3)  1.0 (0.3–3.1) |
| Diabetes Mellitus (uncomplicated) | <1  1–<5  5–<10  ≥10 | 3,372 | 1  1.7 (1.5–1.9)  2.7 (2.2–3.4)  3.1 (1.6–6.1) | 1  1.3 (1.2–1.5)  1.9 (1.5–2.3)  1.9 (1.0–3.9) | 347 | 1  0.6 (0.4–1.1)  1.0 (0.6–1.6)  1.5 (0.7–3.4) | 1  0.6 (0.3–1.0)  0.8 (0.5–1.4)  1.1 (0.5–2.6) |
| Diabetes Mellitus (complicated) | <1  1–<5  5–<10  ≥10 | 4,557 | 1  1.7 (1.6–1.9)  2.7 (2.3–3.1)  3.1 (1.8–5.2) | 1  1.4 (1.2–1.5)  1.9 (1.6–2.2)  1.9 (1.1–3.3) | 53 | 1  1.1 (0.3–4.3)  1.7 (0.4–7.3)  1.4 (0.2–11.3) | 1  1.3 (0.3–5.4)  2.3 (0.5–11.3)  2.0 (0.2–21.2) |
| Hemiplegia | <1  1–<5  5–<10  ≥10 | 5,288 | 1  1.7 (1.6–1.9)  2.7 (2.3–3.0)  3.7 (2.6–5.3) | 1  1.4 (1.2–1.5)  1.9 (1.7–2.2)  2.5 (1.7–3.6) | 3 | -^a^ | -^a^ |
| Moderate/severe kidney disease | <1  1–<5  5–<10  ≥10 | 5,410 | 1  1.7 (1.6–1.9)  2.7 (2.3–3.1)  3.3 (2.3–4.7) | 1  1.4 (1.2–1.5)  1.9 (1.7–2.2)  2.2 (1.5–3.2) | 0 | NA | NA |
| Tumor, leukemia, lymphoma | <1  1–<5  5–<10  ≥10 | 4,770 | 1  1.7 (1.5–1.9)  2.5 (2.2–2.9)  2.9 (2.0–4.1) | 1  1.4 (1.2–1.5)  1.8 (1.6–2.1)  1.9 (1.3–2.7) | 49 | 1  1.3 (0.5–3.4)  -^a^  -^a^ | 1  1.3 (0.4–4.7)  -^a^  -^a^ |
| Moderate/severe liver disease or Metastatic solid tumor | <1  1–<5  5–<10  ≥10 | 5,446 | 1  1.7 (1.6–1.9)  2.6 (2.3–3.0)  3.2 (2.3–4.5) | 1  1.4 (1.3–1.5)  1.9 (1.7–2.2)  2.1 (1.5–3.0) | 0 | NA | NA |
| Hypertension | <1  1–<5  5–<10  ≥10 | 1,027 | 1  1.8 (1.4–2.3)  4.0 (2.0–7.8)  -^a^ | 1  1.1 (0.8–1.5)  2.5 (1.2–5.1)  -^a^ | 1,869 | 1  0.9 (0.7–1.0)  1.2 (1.0–1.5)  1.3 (0.8–2.0) | 1  0.8 (0.7–1.0)  1.0 (0.8–1.2)  1.1 (0.7–1.7) |
| Integrated results | <1  1–<5  5–<10  ≥10 | NA | 1  1.7 (1.7–1.8)  2.6 (2.5–2.7)  3.2 (2.9–3.6) | 1  1.4 (1.4–1.4)  1.9 (1.8–2.0)  2.1 (1.9–2.4) | NA | 1  0.9 (0.8–1.0)  1.2 (1.1–1.4)  1.5 (1.2–1.9) | 1  0.9 (0.8–1.0)  1.1 (0.9–1.3)  1.3 (1.0–1.7) |

OR: odds ratio, CI: confidence interval, PIM: potentially inappropriate medication, NA: not applicable

^a^ Data not available due to the small size for matched controls.
