# Supplementary material for: The Association between Polypharmacy and Dementia: A Nested Case-Control Study Based on a 12-Year Longitudinal Cohort Database in South Korea
Source: PLoS One. 2017 Jan 5;12(1):e0169463. doi: 10.1371/journal.pone.0169463 (PMC5215897; doi:10.1371/journal.pone.0169463)
Supplement: S4 Table — (DOCX) [file pone.0169463.s004.docx]

**S4 Table. Interaction between polypharmacy and predictor variables**

| Category | Predictor variables | p-value |
| --- | --- | --- |
| Drugs showing significant interaction | Anticholinergic drugs | 0.0002 |
|  | H2-receptor antagonists | <0.0001 |
| Drugs showing non-significant interaction | Benzodiazepines (short/intermediate-acting) | 0.1472 |
|  | Benzodiazepines (long-acting) | 0.2672 |
|  | Benzodiazepines (receptor agonist) | 0.1097 |
| Comorbidities showing significant interaction | Hypertension | <0.0001 |
|  | Peripheral vascular disease | 0.0001 |
|  | Cerebrovascular disease | <0.0001 |
|  | Congestive heart failure | 0.0003 |
|  | Hemiplegia | 0.0024 |
|  | Diabetes mellitus (complicated) | 0.0156 |
|  | Diabetes mellitus (uncomplicated) | <0.0001 |
|  | Depression | 0.0002 |
|  | All other mental disorders | <0.0001 |
|  | Chronic obstructive pulmonary disease | <0.0001 |
|  | Peptic ulcer disease | <0.0001 |
|  | Chronic liver disease | <0.0001 |
|  | Group of significant interaction diseases | 0.0003 |
| Comorbidities showing non-significant interaction | Connective tissue disease | 0.0767 |
|  | Schizophrenia and psychotic disorders | 0.1516 |
|  | Behavior disorders due to alcohol | 0.2412 |
|  | Tumor, Leukemia, Lymphoma | 0.3138 |
|  | Moderate/severe kidney disease | 0.4167 |
|  | Metastatic solid tumor | 0.5267 |
|  | Myocardial Infarction | 0.6537 |
|  | Moderate/severe liver disease | 0.8195 |
|  | Delirium | 0.8828 |
|  | Group of non-significant interaction diseases | 0.9506 |
